# Aging affects regrowth of stealthperitoneal dissemination of advanced ovarian cancer: a multicenter retrospective cohort study

**DOI:** 10.1038/s41598-024-66419-w

**Published:** 2024-10-09

**Authors:** Hiroki Fujimoto, Masato Yoshihara, Carmela Ricciardelli, Sho Tano, Shohei Iyoshi, Emiri Miyamoto, Kazumasa Mogi, Maia Hayashi, Sae Hayakawa, Satoshi Nomura, Kazuhisa Kitami, Kaname Uno, Nobuhisa Yoshikawa, Ryo Emoto, Shigeyuki Matsui, Hiroaki Kajiyama

**Affiliations:** 1 Department of Obstetrics and Gynecology, Nagoya University Graduate School of Medicine, Showa-ku, Nagoya, Japan; 2 Discipline of Obstetrics and Gynaecology, Adelaide Medical School, Robinson Research Institute, University of Adelaide, Adelaide, Australia; 3 Center for Medical Education, Nagoya University Graduate School of Medicine, Nagoya, Japan; 4 Department of Obstetrics and Gynecology, Kitasato University Hospital, Sagamihara, Japan; 5 Division of Clinical Genetics, Department of Laboratory Medicine, Lund University Graduate School of Medicine, Lund, Sweden; 6 Department of Biostatistics, Nagoya University Graduate School of Medicine, Nagoya, Japan

**Keywords:** Ovarian cancer, Peritoneal dissemination, Recurrence, Aging, Cancer, Medical research

## Abstract

Ovarian cancer (OvCa) is one of the most lethal gynecological malignancies, and most patients are diagnosed at advanced stage with peritoneal dissemination. Although age at diagnosis is considered an independent prognostic factor, its impact on peritoneal recurrence after combined cytoreductive surgery and chemotherapy is not clear. The objective of this study was to investigate the impact of aging on peritoneal recurrence from stealth dissemination and gain insight of the pathophysiology of OvCa in elderly patients. A total of 243 patients with pT2b-pT3 epithelial ovarian who achieved complete surgery, no-residual tumor at first surgery, were selected to be analyzed the risk of peritoneal seeding and recurrence. We found that age over 65 years was independently associated with an increased risk of peritoneum-specific (PS) recurrence (. Furthermore, pT3 stages and positive ascites cytology also worsen the PS-relapse-free survival. Collectively, our findings suggest that age, especially over 65 years, predicts reduced peritoneum-specific tumor recurrence in patients with advanced ovarian cancer after complete cytoreduction surgery, particularly those with pT3 tumors and positive ascites cytology.

## Introduction

Ovarian cancer (OvCa) has the poorest prognosis and the highest mortality rate among all gynecological malignancies^1^. Generally, the incidence of OvCa increases with increasing age^2,3^, and in Japan the peak age at diagnosis is 57 years^4^. Especially, the most common type of OvCa, high grade serous ovarian cancer (HGSOC), is age-related, and its peak age at diagnosis is 75 years old^4^. As the age increases, more patients are diagnosed with advanced stage disease^2^, and age itself at diagnosis has significant impact on OVCa prognosis^5^. Although it is important to improve the prognosis and quality of life in elderly patients in today's aging society, there is a lack of understanding of pathophysiology of advanced stage OvCa in in the elderly.

Unlike other organs, ovaries are directly exposed to the peritoneal cavity, which shows characteristic metastasis called peritoneal dissemination and omental caking^6^. Furthermore, due to the lack of specific early-stage symptoms, most cases are diagnosed at an advanced stage with peritoneal dissemination^7^. Generally, advanced OvCa patients receive platinum-based chemotherapy after debulking cytoreductive surgery as a first-line treatment^8,9^. Although initial treatment with debulking surgery and following chemotherapy seems effective, over 70% of patients experience recurrence and eventually develop platinum-resistant tumor within 5 years^10^. This may be caused by visually undetectable microscopic metastases in the abdominal cavity, called stealth dissemination, which contributes to the relapse of OvCa^11^.

Recently, the tumor-associated microenvironment (TAM) has attracted many researcher’s attention, and it has been pointed out that TAM in elderly patients may provide a favorable environment for cancer progression^12^. There were some reports of analyzing the prognosis of the elderly patients of OvCa, and advanced age is an independent prognostic factor^2,13–15^. However, there are few studies in the literature that have clinically evaluated the relationship between aging and progression of peritoneal dissemination in detail. Herein, we investigated the effect of aging on peritoneal recurrence from stealth dissemination in OvCa patients with with pT2b-pT3c who received complete tumor resection to provide insight into the pathophysiology of OvCa in elderly patients.

## Methods

### Study participants

We conducted a multicenter retrospective cohort study using the data of the Tokai Ovarian Tumor Study Group, composed of Nagoya University Hospital and its affiliated institutions. Data were collected from the medical charts of the institutions between January 1986 and September 2022. The present study was approved by the Nagoya University Hospital ethics committee (approval number: 2006–0357) in accordance with the guidelines of the Declaration of Helsinki. Data were collected from medical records and clinical follow-up visits; therefore, a written informed consent was waved by the Nagoya University Hospital ethics committee as this study did not include any information that could lead to identification of the participants.

### Study participants

Patients who underwent complete tumor resection in the debulking surgery for pT2b to pT3c epithelial OvCa without distant metastasis were included in the present study. We excluded patients without sufficient clinical data on survival outcome and those who were lost to the follow-up immediately after the surgery. All histopathological slides reviewed by central expert pathologists according to the criteria of the World Health Organization (WHO) classification^16^. Clinical staging was adjusted based on the latest FIGO surgical staging system^17^.

### Surgery, chemotherapy, and follow-up

Primary or interval debulking surgery was performed on all participants. The procedure consisted of complete-staging surgery, including total hysterectomy and bilateral salpingo-oophorectomy with a full peritoneal evaluation with ascites cytology, biopsy, and/or omentectomy, lymphadenectomy, and bowel and/or diaphragm resection if necessarily for complete tumor removal. All patients received chemotherapy as described in our previous study^18^. Patients were followed up with an evaluation of tumor markers and regular pelvic examinations using ultrasonography, magnetic resonance imaging, computed tomography, or positron emission tomography. Tumor recurrence was clinically defined as the development of ascites, a newly detectable lesions with image evaluation, or elevated tumor markers after the complete debulking surgery mainly according to the criteria of the Gynecologic Cancer InterGroup^19,20^ and RECIST guideline (version 1.1)^21^. RFS and overall survival (OS) were defined as the time from the date of the initial surgery until that of the last follow-up, tumor recurrence, or death. Peritoneum-specific-RFS (PS-RFS) was defined as the time from the date of the initial surgery until that of the last follow-up or tumor recurrence to the peritoneum.

### Statistical analysis

Comparisons between groups were performed using the Student’s *t*-test for continuous variables, and the chi-squared or Fisher’s exact test for categorical variables. In analyses of the prognosis of patients, the Kaplan–Meier method was used to compare PS-RFS, RFS, and OS. Differences in survival between two groups were also assessed by the Log-rank test as well as univariate and multivariate Cox regression models. Also, the non-linear association between age, and PS-RFS/RFS was evaluated using generalized additive models with multivariate adjustment. In the subgroup analysis with stratification, the adjusted estimation of the hazard ratio (HR) was also performed by stratifying each variable. Significance was selected as a two-sided P-value < 0.05. All statistical analyses were conducted using IBM SPSS Statistics, Version 28.0 (IBM Corp., Armonk, NY, USA) and R, Version 4.1.3 (https://cran.r-project.org/).

## Results

### Baseline characteristics and survival trend in each age category

**Figure 1:**
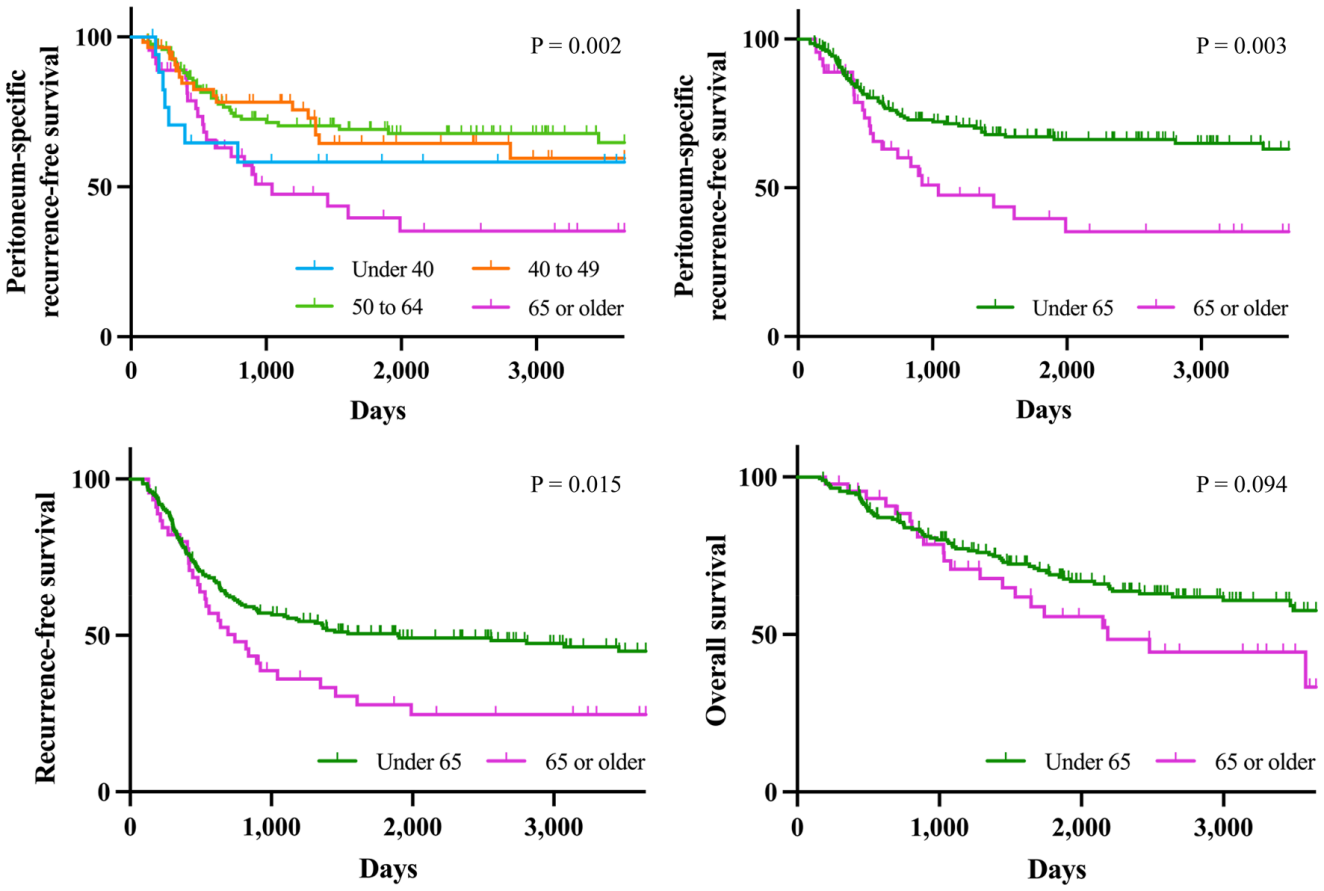
(**A**–**D**) Kaplan–Meier curves for peritoneum-specific recurrence-free (**A**,**B**), recurrence-free (**C**), and overall survival (**D**) in epithelial ovarian cancer patients with each age category (under 45, 40 to 49, 50 to 64, and 65 years or older). P-values were estimated by log rank test.

Among 5268 patients with malignant ovarian tumors, 243 met the study enrolment criteria. The baseline characteristics of this study cohort are shown in Table S1. The distribution of age was shown in Fig. S1; mean and median were 55.1 and 55 years old, respectively. To determine the optimal cut-off point of age category regarding PS-RFS, we performed univariate survival analysis using the Kaplan–Meier method (Fig. 1). When age was categorized into four groups, 39 and younger (adolescent and young adult), 40 to 49 (premenopausal), 50 to 64 (postmenopausal), and 65 and older (elderly), survival was significantly different amongst the patient groups; especially, the 65 and older group that had extremely worse prognosis than the others. As the same trend was confirmed in RFS and OS (not significant), we divided the original cohort into two age groups (< 65 years and ≥ 65 years). The majority of patients with recurrent tumors had peritoneal metastasis (Fig. S1). In patients who developed recurrent tumor on the peritoneum, there was no significant difference in the PS-recurrence-free interval between the two groups.

### Multivariate analysis of survival outcomes

Baseline characteristics of the two groups are-described in Table 1. More patients in the older group were diagnosed with pT3 stage and had serous histology compared to the younger group. In contrast, retroperitoneal lymph node metastasis was more frequently detected in the younger group. Univariate and multivariate analyses using a Cox regression hazard model for PS-RFS and RFS are shown in Table 2 and Table S2. We found that age over 65 years was independently associated with increased risk of PS- recurrence. In addition, pT3 stages (compared to pT2) and positive ascites cytology also worsed the PS-RFS, similar to RFS.

**Table 1:** Baseline characteristic of patients in the two groups.

Category	< 65 years (n = 198)	65 ≤ years (n = 45)	P-value
pT stage, n (%)			
pT2b	80 (40.4)	9 (20.0)	0.032
pT3a	21 (10.6)	3 (6.7)	
pT3b	36 (18.2)	12 (26.7)	
pT3c	61 (30.8)	21 (46.7)	
pN stage, n (%)			
pN0	82 (41.4)	16 (35.6)	0.011
pNX	72 (36.4)	36 (57.8)	
pN1	44 (22.2)	3 (6.7)	
Histology, n (%)			
Serous	96 (48.5)	32 (71.1)	0.044
Clear-cell	64 (32.3)	9 (20.0)	
Mucinous	6 (3.0)	0 (0.0)	
Endometrioid	32 (16.2)	4 (8.9)	
Hysterectomy, n (%)	186 (93.9)	42 (93.3)	0.549
CA-125, IU/mL	1114.2 (2102.3)	1520.7 (2530.0)	*0.261
Positive ascites cytology, n (%)	100 (50.5)	29 (64.4)	0.091
Chemotherapy, n (%)	198 (100.0)	45 (100.0)	n.a

Data are presented as mean (standard deviation) or proportion (%).

Student’s *t*-test, chi-square test, or Fisher's exact test was used as appropriate.

SD, standard deviation; CA, cancer antigen.

*Logarithmically transformed when analyzed.

**Table 2:** Cox regression analysis for assessing factors associated with peritoneum specific-recurrence-free survival (n = 243).

Categories	Univariate		Multivariate	
	HR (95%CI)	P value	HR (95%CI)	P value
Age categories				
< 65 years	Reference		Reference	
65 ≤ years	2.031 (1.252–3.295)	0.004	1.887 (1.129–3.155)	0.015
pT stage				
pT2b	Reference		Reference	
pT3a	3.023 (1.427–6.402)	0.004	2.755 (1.285–5.907)	0.009
pT3b	2.108 (1.099–4.042)	0.025	1.524 (0.772–3.007)	0.225
pT3c	2.837 (1.608–5.006)	< 0.001	1.890 (1.014–3.523)	0.045
pN stage				
pN0/X	Reference		Reference	
pN1	1.598 (0.955–2.676)	0.075	1.314 (0.746–2.316)	0.344
Histology				
Non-serous	Reference		Reference	
Serous	2.004 (1.262–3.180)	0.003	1.335 (0.797–2.235)	0.272
Hysterectomy	0.626 (0.288–1.359)	0.236	0.489 (0.221–1.078)	0.076
*CA-125, IU/mL	1.166 (1.023–1.330)	0.022	1.087 (0.941–1.256)	0.257
Positive ascites cytology	2.035 (1.293–3.204)	0.002	1.609 (1.005–2.577)	0.048

HR, hazard ratio; CA, cancer antigen.

*Logarithmically transformed when analyzed.

**Figure 2:**
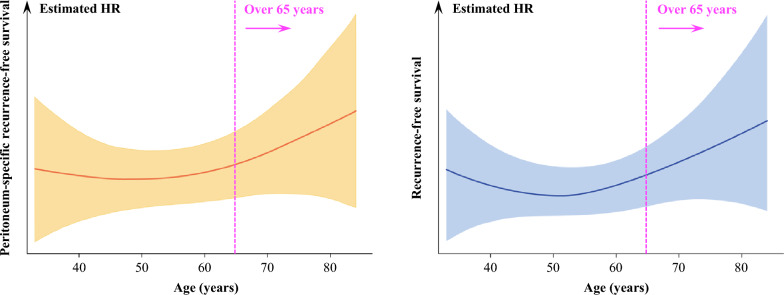
Spline models of hazard ratio regarding peritoneum-specific recurrence-free (**A**) and recurrence-free survival (**B**) for age, adjusted by pT stage, pN stage, histology, hysterectomy, CA-125 level, and result of cytology.

We also investigated the impact of aging for PS-RFS, recognizing age as a continuous variable. Estimated HR in a spline model with multivariate adjustment is shown in Fig. 2. The hazard of peritoneal recurrence rose from the age of 65 years, of which trend was similar in that of all recurrence. Collectively, the findings suggested that aging after 65 years increase the risk of tumor recurrence including at the peritoneum.

### Subgroup analysis

**Figure 3:**
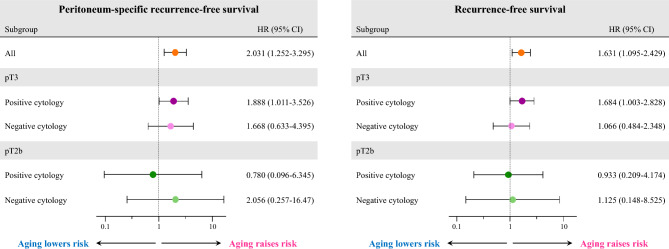
Estimation of the hazard ratio of peritoneum-specific recurrence-free (**A**) and recurrence-free survival (**B**) with 95% confidence interval for age category of 65 years or older in each subgroup.

We also explored the impact of age on the tumor recurrence among subgroups of tumor stages and ascites cytology. We estimated the HR of PS recurrence and all recurrence for age over 65 years in the four subgroups: pT2b/cytology-positive, pT2b/cytology-negative, pT3/cytology-positive, and pT3/cytology-negative. Age over 65 years had a significant negative impact on PS-RFS in patients with both pT3 tumors and positive ascites cytology. In contrast, the age category did not deteriorate PS-RFS in the other subgroups, where the trend was similar to RFS (Fig. 3). Overall, these results suggest that aging, especially over 65 years, was significantly associated with a worse prognosis and increased risk of peritoneum-specific tumor recurrence after complete tumor resection, particularly in those patients with pT3 tumors and positive ascites cytology.

## Discussion

In this study, PS-RFS as well as RFS is significantly reduced in OvCa patients diagnosed at 65 years or older, suggesting that age over 65 years independently increased risk of developing recurrent tumor from stealth dissemination of OvCa. This is the first report assessing the relationship between aging and tumor recurrence in advanced stage OvCa patients following complete surgical resection.

This result is consistent with previous reports analyzing prognosis of elderly patients with advanced OvCa^5,22^. There are some studies that linked aging and tumor progression. For example, Teramukai S, et al., reported that advanced age over 70 years old was one of the significant prognostic indicators of OS in advanced OvCa^23^. Our results indicated that age of 65 or older was associated with increased hazard of peritoneal recurrence as well as stage and positive ascites cytology. Consecutively visualized HR for PS-RFS also demonstrated the negative impact of aging for peritoneal recurrence, particularly those with pT3 tumors and positive ascites cytology. Collectively, these findings suggested that aging as one of the indicators of host environment potentially affected behavior of OvCa especially in the peritoneum.

The difference in survival outcomes between the age categories despite receiving similar treatments may be explained by changes in tumor microenvironment associated with aging as “the seed and soil hypothesis” proposed by Dr. Stephan Paget^24^. In other words, the negative impact of aging as a “soil” can enhance the metastatic potential of the “seed” of OvCa cells in the peritoneal environment.

**Figure 4:**
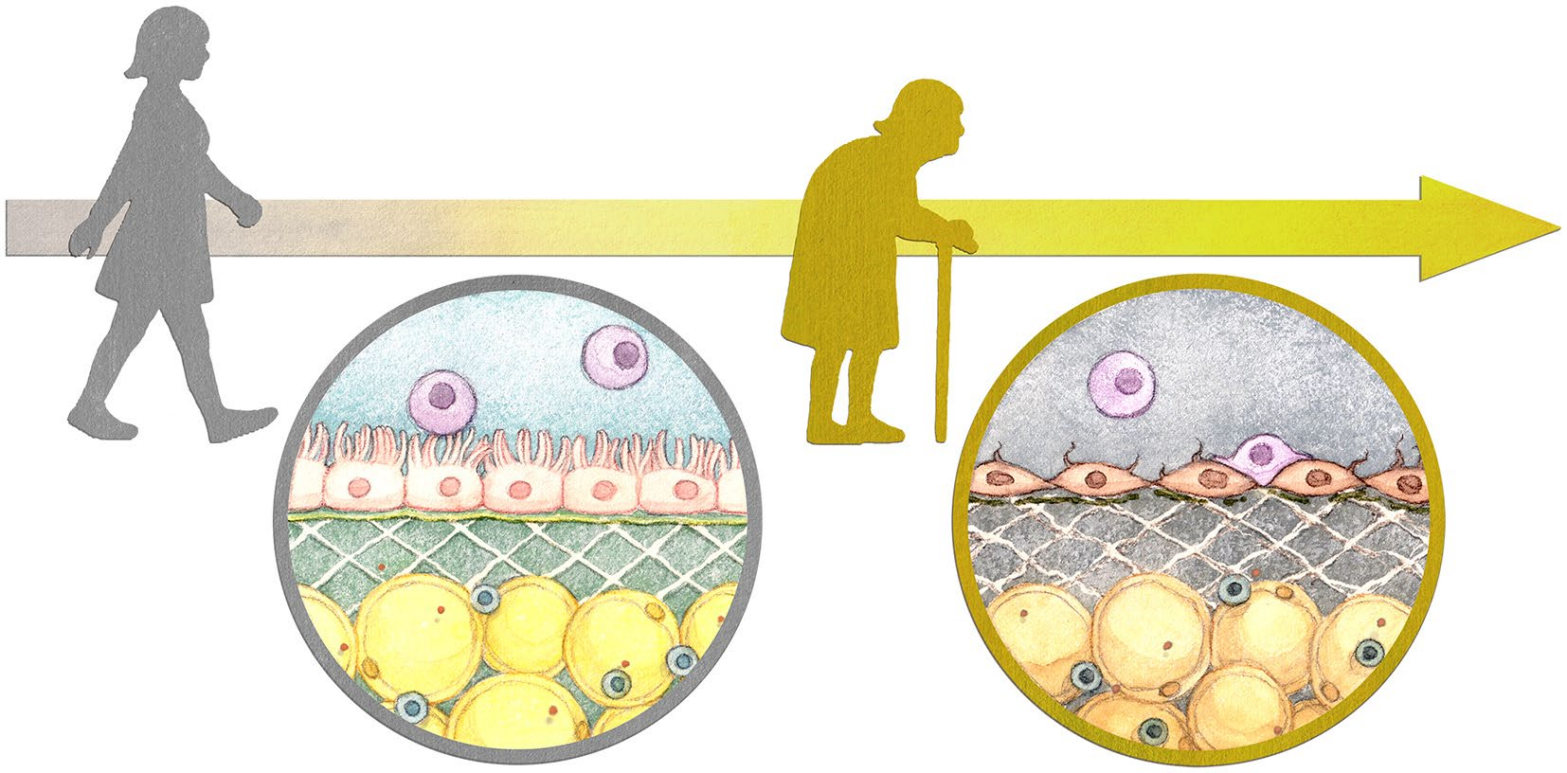
Schematic image of the impact of aging on regrowth of stealth peritoneal dissemination.

The molecular interactions between tumor cells and host cells in peritoneal dissemination are gradually becoming clear from many studies of various types of cancers, including ovarian cancer. In the treatment of advanced ovarian cancer, tumor cells are drastically reduced in primary debulking surgery, and platinum-based chemotherapy is performed to reduce the number of invisible residual tumor cells. However, as many cases of advanced OvCa experience recurrence, how these few remaining platinum-resistant tumor clones after the initial treatment interact with the host microenvironment and recur intraperitoneally is a pivotal question to improve the prognosis of patients suffering from advanced OvCa. As shown in the peritoneal metastatic cascade, the interaction of tumor cells with various types of host cells may result in changes in the peritoneal microenvironment, such as vascularization, enabling tumor cells that have survived the surgical and chemotherapy interventions to sprout again intraperitoneally^25^. In fact, one report showed that increased age was associated with higher percentage of intraperitoneal metastasis and it also indicated that senescent peritoneal mesothelium creates a niche for OvCa metastases^26^. Several in vivo studies also showed that higher efficacy of intraperitoneal dissemination in aged mice compared to younger mice^27,28^. Another report showed that the intraperitoneal injection of OvCa cells with senescent human peritoneal mesothelial cells into the mice promoted further metastasis^29^. Interestingly, the comparison of collagen fiber orientation in the omentum between young and aged mice by second harmonic generation (SHG) showed more sparse collagen fiber network in aged mice, which might provide the scaffold for tumor invasion^12,27^. These results may suggest that aging creates the more favorable peritoneal microenvironment for progression of OvCa (Fig. 4).

Furthermore, recent studies indicate that biological aging is strongly associated with accumulation of senescent cells, which secretes the senescence-associated secretory phenotype (SASP) that have various downstream effects, such as carcinogenesis^30,31^. In gastric cancer, SASP secreted by aging cancer-associated fibroblasts (CAFs) has been shown to create a TAM with the Janus Kinases (JAKs)/ Signal transducer and activator of transcription protein (STATs) axis for further progression^32^. In addition, the extracellular matrix (ECM) from aged mice altered the expression pattern of cancer-related genes in a breast cancer model, transforming them to more invasive tumors^33^. Furthermore, aging also weakens the ability of the immune system to eliminate tumor cells, and inflammation relating to aging is associated with enhanced carcinogenesis and tumor progression^34^. These aging associated immune dysfunction^35^, described as “immune-senescence” and chronic inflammation, described as “inflammaging”, are strongly associated with tumor progression^34,36–38^. Although similar mechanism may exist in peritoneal tumor microenvironment, it has not yet been demonstrated in OvCa. Furthermore, the large volume of tumors in the peritoneal dissemination might contain varieties of clones with different biological properties, enabling different mechanisms of intraperitoneal dissemination^39^. Interestingly, recent technology, such as spatial transcriptome analysis in OvCa, indicates that HGSOC cells interact with diverse surrounding cells, such as fibroblast and immune cells, and modulate their local microenvironment^40^. Although there is still room for further development in this field, our study suggests that the tumor microenvironment changes with aging, and further molecular mechanistic mechanisms are expected to be elucidated in the future.

A limitation of present study is the small number of cases involved. In addition, the study does not include the impact of complications and frailties that may occur with aging. In a report comparing OvCa treatment in older and younger patients, it indicates that there was a significant difference in surgical completion and the number of patients who underwent chemotherapy depending on comorbidities and performance status (PS)^2,13,15^. It has also been suggested that older patients tend to have less opportunities to receive standardized treatment^14^. In addition, the existence of unknown confounding factors cannot be eliminated. However, we believe this study still has a significance that suggested the influence of aging in advanced OvCa since we analyzed clinical information from multiple affiliated institution with the central pathological review system. Further analysis is necessary to determine the effect of aging on recurrence, which could contribute to improving the prognosis of the elderly patients with advanced OvCa.

In conclusion, age, especially over 65 years, was significantly associated with a worse prognosis and reduced peritoneum-specific tumor recurrence in advanced OvCa patients even after complete tumor resection, particularly those with pT3 tumors and positive ascites cytology. Factors including age of the host is an important environmental factorthat should be also recognized as determining survival outcome in OvCa.

## Supplementary Information

Supplementary Figure S1.

Supplementary Table S1.

Supplementary Table S2.

## Data Availability

The datasets generated and/or analyzed during the current study are not publicly available because it contains patients’ clinical information but are available from the corresponding author on reasonable request.
