# Supplementary material for: Aging affects regrowth of stealthperitoneal dissemination of advanced ovarian cancer: a multicenter retrospective cohort study
Source: Sci Rep. 2024 Oct 9;14:23537. doi: 10.1038/s41598-024-66419-w (PMC11479624; doi:10.1038/s41598-024-66419-w)
Supplement: Supplementary file 1 — Supplementary Figure S1. [file 41598_2024_66419_MOESM1_ESM.pptx]

## Slide 1
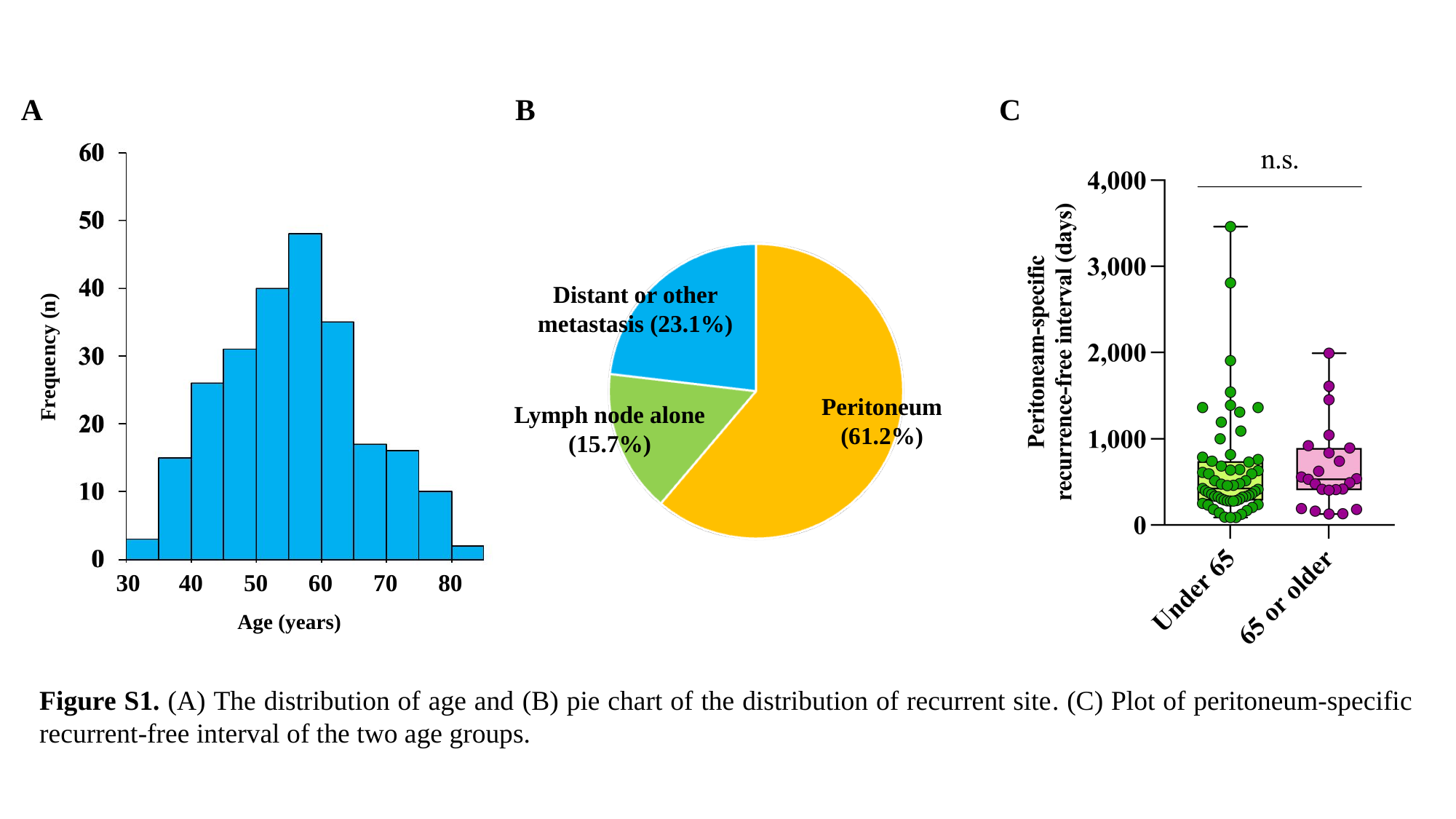

A
B
C
Distant or other
metastasis (23.1%)
Frequency (n)
Peritoneum
(61.2%)
Lymph node alone
(15.7%)
30
40
50
60
70
80
Age (years)
Figure S1. (A) The distribution of age and (B) pie chart of the distribution of recurrent site. (C) Plot of peritoneum-specific recurrent-free interval of the two age groups.
