# Supplementary material for: Aging affects regrowth of stealthperitoneal dissemination of advanced ovarian cancer: a multicenter retrospective cohort study
Source: Sci Rep. 2024 Oct 9;14:23537. doi: 10.1038/s41598-024-66419-w (PMC11479624; doi:10.1038/s41598-024-66419-w)
Supplement: Supplementary file 2 — Supplementary Table S1. [file 41598_2024_66419_MOESM2_ESM.docx]

**Table S1.** Baseline characteristic of patients in this study cohort.

| **Categories** | **Epithelial OvCa (n =243)** |
| --- | --- |
| Age, years (SD) | 55.1 (10.7) |
| Age categories, n (%) |  |
| ≤39 years | 18 (7.4) |
| 40–49 years | 57 (23.5) |
| 50–64 years | 123 (50.6) |
| 65≤ years | 45 (18.5) |
| pT stage, n (%) |  |
| pT2b | 89 (36.6) |
| pT3 | 154 (63.4) |
| pN stage, n (%) |  |
| pN0/NX | 196 (80.7) |
| pN1 | 47 (29.3) |
| Histology, n (%) |  |
| Serous | 128 (52.7) |
| Clear-cell | 73 (30.0) |
| Mucinous | 6 (2.5) |
| Endometrioid | 36 (14.8) |
| Hysterectomy, n (%) | 228 (93.8) |
| CA-125, IU/mL | 1189.5 (2187.9) |
| Positive ascites cytology, n (%) | 129 (53.1) |
| Chemotherapy, n (%) | 243 (100.0) |

Abbreviations: OvCa, ovarian cancer; SD, standard deviation; CA, cancer antigen.
