# Supplementary material for: Aging affects regrowth of stealthperitoneal dissemination of advanced ovarian cancer: a multicenter retrospective cohort study
Source: Sci Rep. 2024 Oct 9;14:23537. doi: 10.1038/s41598-024-66419-w (PMC11479624; doi:10.1038/s41598-024-66419-w)
Supplement: Supplementary file 3 — Supplementary Table S2. [file 41598_2024_66419_MOESM3_ESM.docx]

**Table S2.** Cox regression analysis for assessing factors associated with recurrence-free survival (n = 243).

|  | **Univariate** | | **Multivariate** | |
| --- | --- | --- | --- | --- |
| **Categories** | **HR (95%CI)** | **P value** | **HR (95%CI)** | **P value** |
| Age categories |  |  |  |  |
| <65 years | reference |  | reference |  |
| 65≤ years | 1.631 (1.095–2.429) | 0.016 | 1.563 (1.026–2.382) | 0.038 |
| pT stage |  |  |  |  |
| pT2b | reference |  | reference |  |
| pT3a | 2.549 (1.394–4.663) | 0.002 | 2.307 (1.253–4.248) | 0.007 |
| pT3b | 2.112 (1.285–3.472) | 0.003 | 1.674 (0.992–2.825) | 0.054 |
| pT3c | 2.637 (1.700–4.089) | <0.001 | 1.940 (1.194–3.151) | 0.007 |
| pN stage |  |  |  |  |
| pN0/X | reference |  | reference |  |
| pN1 | 1.862 (1.264–2.743) | 0.002 | 1.509 (0.984–2.315) | 0.059 |
| Histology |  |  |  |  |
| Non-serous | reference |  | reference |  |
| Serous | 1.548 (1.092–2.193) | 0.014 | 1.007 (0.681–1.490) | 0.971 |
| Hysterectomy | 1.056 (0.493–2.260) | 0.889 | 0.992 (0.408–1.908) | 0.750 |
| CA-125, IU/mL* | 1.143 (1.032–1.267) | 0.011 | 1.087 (0.972–1.217) | 0.144 |
| Positive ascites cytology | 1.756 (1.240–2.488) | 0.002 | 1.440 (1.003–2.069) | 0.048 |

Abbreviations: HR, hazard ratio; CA, cancer antigen.

* Logarithmically transformed when analyzed.
